# Reidentification of hybridization events with transcriptomic data and phylogenomic study in Seabuckthorn

**DOI:** 10.1038/s41598-025-09923-x

**Published:** 2025-07-06

**Authors:** Hui Zhang, Zhiqi Wang, Xue Su, Dong Han, Lujie Yang, Ying Zhang, Jing Fang, Jingyuan Wang, Kun Sun

**Affiliations:** College of Life Sciences, Northwest Normal University, Lanzhou, China

**Keywords:** Sea Buckthorn, Transcriptome, SNP calling, F1 hybrid identification, Phylogenomics, Genetics, Plant sciences, Systems biology

## Abstract

Natural hybridization in sea buckthorn (*Hippophae spp*.) is well documented. While the parental species involved in these events have been identified, distinctions between F1 hybrids and later-generation (Fn) hybrids remain insufficiently explored, and their genetic compositions are not yet fully understood. In this study, we employed transcriptomic data and reference genomes to identify Fn hybrids in two natural hybrid populations, confirming eight individuals—including *H*. *goniocarpa* Lian. X. L. Chen et K. Sun and four members of a hybrid swarm from Qinghai, China—as F1 hybrids. These findings support the hypothesis that *H. goniocarpa* is not a distinct species, but rather an F1 hybrid within the genus. Additionally, we discuss limitations specific to SNP calling from transcriptomic data—such as allele-specific expression and low transcript abundance—which may lead to the misclassification of heterozygous sites as homozygous. Finally, we constructed the first phylogenomic tree of the *Hippophae* genus using transcriptomic data and performed a comparative analysis of interspecific relationships based on SNP and indel markers derived from the same dataset.

## Introduction

Since Linnaeus introduced binomial nomenclature and established the foundations of modern taxonomy, organisms have primarily been classified based on phenotypic traits—for example, the reproductive organs of plants—which has long facilitated their recognition and differentiation^1^. However, advances in sequencing technologies have revolutionized species classification by enabling the construction of phylogenomic trees from gene sequences. Molecular approaches provide a more powerful and precise framework for evolutionary research than traditional phenotypic methods^2^.

High-quality transcriptome data generated by second-generation sequencing supports comprehensive analyses of gene sequences, including phylogenetic reconstruction and single nucleotide polymorphism (SNP) calling^3,4^. By leveraging available reference genomes alongside transcriptomic data, researchers can identify vast numbers of SNPs and insertion–deletion polymorphisms (INDELs) that characterize genomic features^5^. These abundant molecular markers offer robust evidence for comparing genomes across different species^6^.

During the early period of the modern evolutionary synthesis (1924–1950), interspecific hybridization was generally viewed as rare and of minimal evolutionary significance—a perspective shaped largely by studies of animal systems with strong reproductive barriers. In contrast, botanists have long documented natural hybrids, with floristic surveys indicating that approximately 10% of plant species engage in hybridization^7^. Hybridization may result either in the emergence of new species or remain confined to the F1 generation. Although polyploid hybrid species are relatively common, the formation of homoploid hybrids is considered rarer^8^; in some cases, reproductive isolation or the maladaptation of later-generation hybrids causes these crosses to persist only as F1 individuals.

In recent years, the origin of diploid hybrids has attracted increasing attention. For instance, Liu et al.^9^ investigated the formation of diploid hybrids using variations in nuclear and chloroplast DNA sequences combined with approximate Bayesian computation and ecological niche modeling. The accurate identification of hybrid offspring is critical for applications in species conservation, germplasm management, and crop breeding^10^. Historically, hybrids were identified by the simultaneous presence of parental phenotypes; however, this method is limited by the availability of distinguishing traits and its inability to differentiate F1 hybrids from backcrosses. The subsequent development of isozyme molecular markers provided a more refined approach: by determining whether two distinct allozymes are produced at a specific gene locus, researchers can assess heterozygosity at that locus^11^. In F1 hybrids, loci exhibiting fixed differences between parental species are expected to be heterozygous^12^.

Transcriptome data have traditionally been used for differential gene expression analysis and, in some cases, for SNP calling and related applications. However, SNP calling from transcriptome data should be distinguished from that based on resequencing data, as the identification of heterozygous sites may be influenced by allele-specific expression and the low expression of certain genes.

Sea buckthorn (genus *Hippophae*, Family *Elaeagnaceae*) is a deciduous shrub or tree valued not only for its nutritious fruits but also for its role as a pioneer species in soil improvement, wind and sand control, and soil and water conservation, rendering it of considerable ecological and economic importance^13^. The Tibetan Plateau and its adjacent regions—including the Himalayas and Hengduan Mountains—are recognized as the ancestral habitat of *Hippophae*. Following its origin, sea buckthorn is believed to have migrated and evolved along two primary routes: one toward the Loess Plateau and North China, and the other from Central Asia toward Europe. This migration, influenced by interactions with diverse landforms and climates, led to the emergence of different species and subspecies^14^. According to the classification system proposed by Lian et al., the genus comprises six species and seventeen subspecies (Table 1), with the Tibetan Plateau and neighboring areas (e.g., Xinjiang, Gansu, Sichuan, Yunnan) harboring six species and thirteen subspecies, while four subspecies are distributed in Europe (*H*. *rhamnoides subsp*. *rhamnoides*, *H. subsp. fluviatilis*, *H*. *rhamnoides subsp*. *carpatica*, and *H*. *rhamnoides subsp*. *caucasia*).

**Table 1:** Systematic classification of sea Buckthorn genus.

Section 1. Hippophae	Section 2. Gyantsenses Lian
*H*. *rhamnoides Linn*.	*H*. *goniocarpa* Lian. X. L. Chen et K. Sun
ssp. *sinensis* Rousi	ssp. *litangensis* Lian et. X. L. Chen
ssp. *wolongensis* Y. S. Lian, K. Sun & X. L. Chen	ssp. *goniocarpa*
ssp. *yunnanensis* Rousi	*H*. *gyantsensis* (Rousi) Lian
ssp. *turkestaniea* Rousi	ssp. *linearifolia*
ssp. *mongolica* Rousi	ssp. *gyantsensis*
ssp. *caucasia* Rousi	*H*. *neurocarpa* S. W. Liu et T. N. He
ssp. *carpatica* Rousi	ssp. *neurocarpa*
ssp. *rhamnoides*	ssp. *Stellatopilosa* Lian et X. L. Chen
ssp. *fluviatilis* Van Soest	*H*. *tibetana* Schlecht
*H*. *salicifolia* D.Don	ssp. *yadongensis*
	ssp. *tibetana*

Notably, *H*. *goniocarpa*, discovered in Rixu Village, Qinghai Province, China, is suspected to have originated through homoploid hybridization and has been identified as a hybrid of *H*. *rhamnoides subsp*. *sinensis* and *H*. *neurocarpa*^15^. In addition, a hybrid descendant exhibiting characteristics of both *H*. *neurocarpa* and *H*. *tibetana* has been identified in the Tibet region^16^.

In this work, we identify the hybrid F1 generation using transcriptome data and provide an analysis of the challenges and limitations related to SNP calling at heterozygous sites in transcriptomic datasets. Furthermore, we constructed a robust phylogenomic tree that elucidates the evolutionary relationships among the five known sea buckthorn species using single-copy orthologs derived from transcriptome data. A comparative genomic analysis based on SNPs and INDELs was then performed across seven sea buckthorn taxa—including *H*. *rhamnoides subsp*. *sinensis*, *H*. *rhamnoides subsp*. *mongolica*, *H*. *rhamnoides subsp*. *yunnanensis*, *H*. *tibetana*, *H*. *salicifolia*, *H*. *gyantsensis*, and *H*. *neurocarpa*—to further characterize their genomic differentiation and evolutionary history.

## Materials and methods

### Materials

For this study, transcriptome data were obtained from multiple sources. In addition to generating new RNA-Seq data from sea buckthorn individuals sampled from various elevations in northwest China (see Tables 2 and 3 for detailed sampling information), transcriptome datasets for *H*. *gyantsensis*, *H*. *salicifolia*, *H*. *rhamnoides subsp*. *yunnanensis*, and *H*. *rhamnoides subsp*. *mongolica* were downloaded from the NCBI Sequence Read Archive (SRA) using SRAtoolkit v3.0.1. Overall, 71 transcriptome datasets from our laboratory (collected in two independent batches) and four datasets from the SRA (totaling 75 datasets) were processed. Adapter sequences and low-quality reads were removed using TrimGalore v0.6.7, and the resulting high-quality “clean” reads were used for all downstream analyses.

**Table 2:** Transcriptome sequencing sampling information. The H. sis2 group consists of six individuals: H. sis2_1, H. sis2_2, and H. sis2_3 are females, while H. sis2_4, H. sis2_5, and H. sis2_6 are males. The H. neu4 group also contains six individuals, with H. neu4_1, H. neu4_2, and H. neu4_3 being females, and H. neu4_4, H. neu4_5, and H. neu4_6 being males. All other individuals are female.

H.sis	elevation	location	H. gon	elevation	location	H. neu	Elevation	Location
H. sis5_1	3188	Ri Xu Village	H. gon_1	3188	Ri Xu	H. neu2_1	3188	Ri Xu Village
H. sis5_2			H. gon_2		Village	H. neu2_2		
H. sis5_3			H. gon_3			H. neu2_3		
H. sis4_1	2934	Mengyuan County Haomen Bridge	H. gon_4					
H. sis4_2						H. neu7_1	3594	Dalang Village
H. sis4_3						H. neu7_2		
						H. neu7_3		
H. sis3_1	2563	Zhangye Mati Temple						
H. sis3_2						H. neu4_1	3332	Daquan Village
H. sis3_3								
H. sis2_1	2267	Zhangye Dayekou Nature Reserve				H. neu4_2		
H. sis2_2						H. neu4_3		
H. sis2_3						H. neu4_4		
H. sis2_4						H. neu4_5		
H. sis2_5						H. neu4_6		
H. sis2_6								
H. sis1_1	1458					H. neu1_1	3024	Bianma Village
		Zhangye 312 National Road Heiheqiaotou						
H. sis1_2						H. neu1_2		
H. sis1_3						H. neu1_3		

**Table 3:** Transcriptome sequencing sampling information.

H. tib	Elevation	Location	Hybrid offspring	Elevation	Location	H. neu	Elevation	Location
H. tib4_1	4146	Da Ri	TN_D1	4146	Da Ri	H. neu9_1	4146	Da Ri
H. tib4_2			TN_D2			H. neu9_2		
H. tib4_3			TN_D3			H. neu9_3		
			TN_D4					
H. tib3_1	4066	Dong Ri Si				H. neu8_1	3895	Guoluo Huashi Gorge
H. tib3_2						H. neu8_2		
H. tib3_3						H. neu8_3		
H. tib2_1	3533	Da Lang Village				H. neu6_1	3533	Da Lang Village
H. tib2_2						H. neu6_2		
H. tib2_3						H. neu6_3		
H. tib2_4						H. neu5_1	3352	Bing Gou
H. tib2_5						H. neu5_2		
H. tib2_6						H. neu5_3		
H. tib1_1	3188	Ri Xu Village				H. neu3_1	3188	Ri Xu Village
H. tib1_2						H. neu3_2		
H. tib1_3						H. neu3_3		

### Methods

#### SNP calling and gene expression analysis

Clean reads were aligned to the sea buckthorn reference genome^17^ using HISAT2 v2.2.1^18^. Single nucleotide polymorphisms (SNPs) were then identified using the Genome Analysis Toolkit (GATK) v4.2.3.0^19^. The resulting VCF files were processed with vcfR v1.14.0^20^ to extract genotype and sequencing depth information. The chromosomal distribution of SNPs was visualized with CMplot v4.5.0^21^. For further processing, bcftools v1.17^22^ was used to extract variant calls, and these VCF files were converted to BED format using Bedops v2.4.41^23^; genes harboring SNPs were then identified using bedtools v2.29.1^24^.

For gene expression analysis, BAM files generated by HISAT2 were processed with featureCounts v2.0.1^25^ to count the number of reads mapped to each gene. The resulting count data were normalized using DESeq2 v1.32.0^26^, and subsequent analyses—including principal component analysis (PCA) and hierarchical clustering (heatmap analysis)—were performed to explore expression patterns across samples.

#### Construction of the phylogenomic tree

For four sea buckthorn taxa (*H*. *rhamnoides subsp*. *mongolica*, *H*. *rhamnoides subsp*. *yunnanensis*, *H*. *salicifolia*, and *H*. *gyantsensis*), de novo transcriptome assemblies were generated using Trinity v2.8.5^27^. For the remaining individuals, transcriptome reads from multiple samples collected at the same location were merged to produce a single, representative assembly for each sampling point.

From each assembly, the longest transcript per gene was selected as the “unigene.” Coding sequences (CDSs) were predicted using TransDecoder v5.5.0. Single-copy orthologous genes were then identified using OrthoFinder v2.5.4^28^. The protein sequences corresponding to these orthologs were aligned using MUSCLE v5.1^29^ and subsequently trimmed with Gblocks v0.91b^30^. A phylogenetic tree was constructed from the trimmed protein alignments using RAxML v8.2.12^31^. The CDSs corresponding to the protein alignments were extracted and aligned at the codon level with PRANK v170427^32^; the codon alignments were further trimmed using trimAl v1.4.rev15^33^. Finally, divergence times were estimated using MCMCtree v4.10.6^34^.

## Results

### Identification of hybrid offspring in sea Buckthorn

#### Identification of G_R1 individuals

SNP calling was performed on four *H*. *goniocarpa*, three *H*. *rhamnoides ssp.*. *sinensis*, and three *H*. *neurocarpa* individuals, all collected from the same sampling site. From the SNP and INDEL data of the *H. rhamnoides ssp. sinensis* and *H*. *neurocarpa* samples, we identified 320,029 loci that were consistently homozygous and genotype-consistent within each species, yet exhibited distinct genotypes between species. If the *H*. *goniocarpa* individual represents a hybrid F1 generation, its genome should exhibit heterozygosity at these loci.

**Fig. 1:**
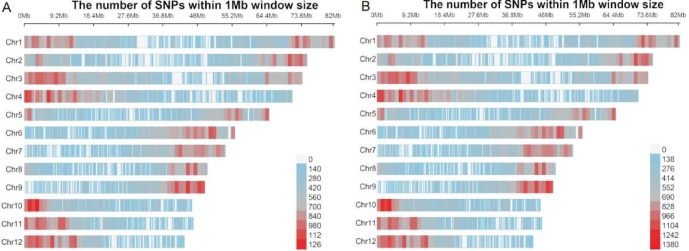
(**A**) Heterozygous SNP distribution of G_R1 individuals of *H*. *goniocarpa.* (**B**) Distribution of 285,831 randomly extracted SNPs from G_R1 individuals of *H*. *goniocarpa*.

In the *H*. *goniocarpa* individual designated H. gon_1, approximately 285,831 of these loci (89.31%) were heterozygous, while the remaining 34,198(10.69%) were homozygous for one of the parental alleles. Visualization of the heterozygous SNPs and INDELs across all twelve chromosomes revealed an even distribution, which was consistent with the pattern observed in a randomly sampled set of variants (Fig. 1). These findings indicate that no genetic recombination has occurred, confirming that H. gon_1 is a hybrid F1 individual.

**Fig. 2:**
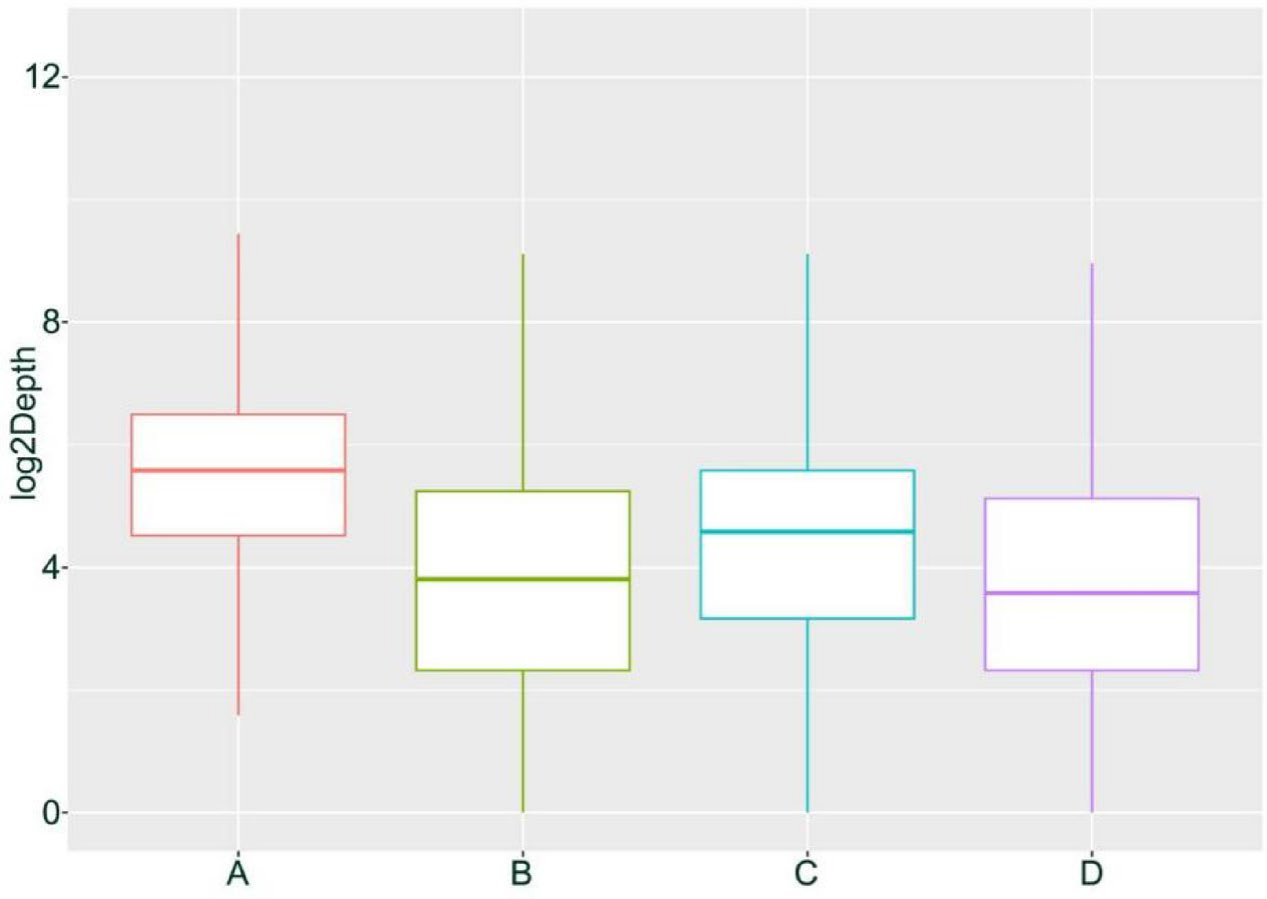
Depth distribution of SNPs and INDELs in four groups of G_R1 individuals of *H*. *goniocarpa*.

**Fig. 3:**
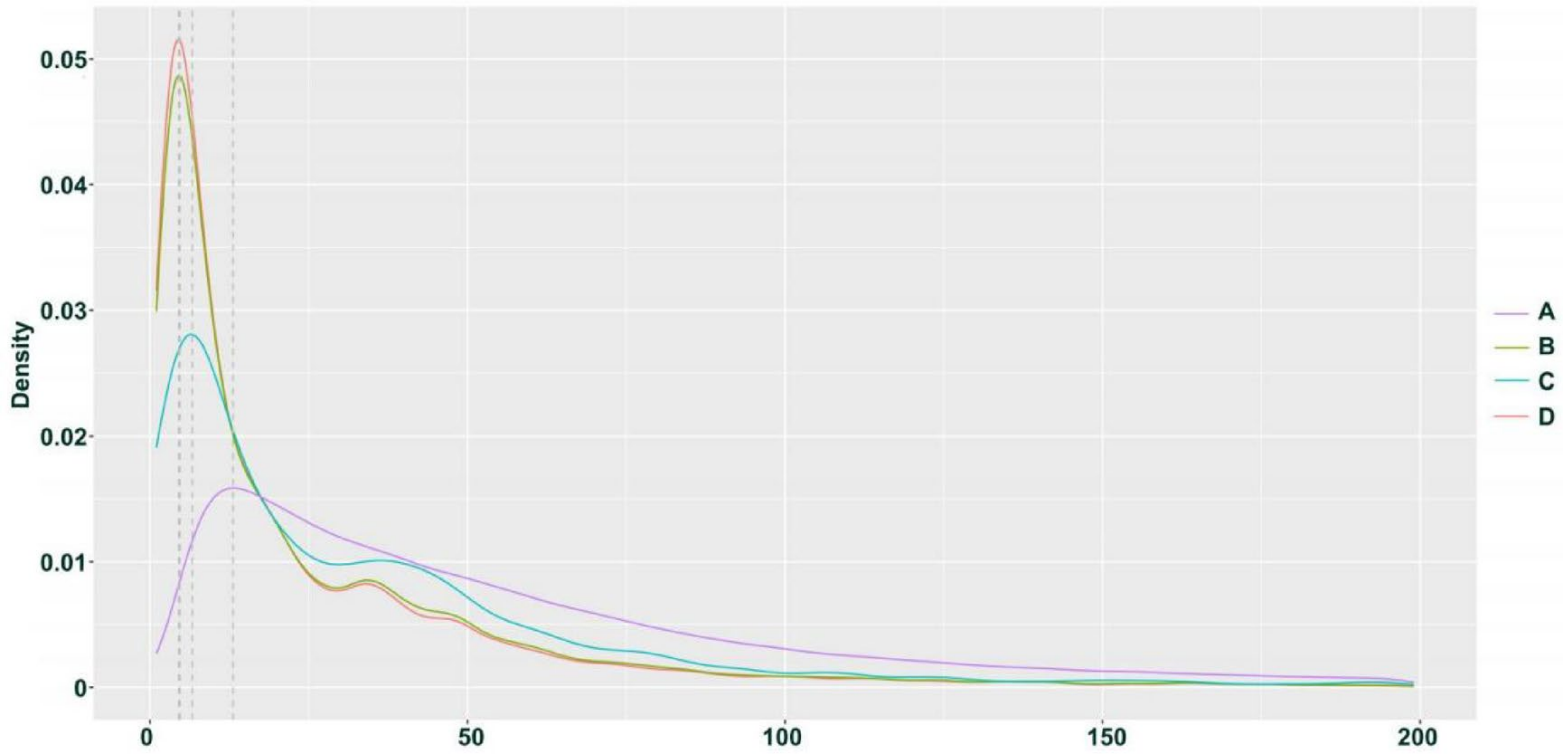
Density distribution of four groups of SNP depths in H. gon_1 individuals of *H*. *goniocarpa*.

We hypothesize that the observed homozygous loci may result from misclassification due to allele-specific expression (ASE) and low expression levels of certain genes. Based on previous research, we speculate that if the four individuals represent F1 hybrids, then their parents can be inferred with some confidence: the male parent is likely *H*. *neurocarpa*, and the female parent is likely *H*. *rhamnoides ssp.*. *sinensis*. we classified SNPs and INDELs expected to be heterozygous in each hybrid into four groups. Group A includes loci exhibiting normal heterozygosity. Group B consists of loci misclassified as homozygous, which can be further divided into two subgroups: Group C, comprising loci that are consistently homozygous across all four individuals and share the same genotype, likely due to ASE; and Group D, consisting of loci that were likely misclassified due to low expression levels, where one allele was not detected during sequencing, resulting in heterozygous sites being erroneously called as homozygous. The sequencing depth distribution of these four groups in H. gon_1 individuals is shown in Figs. 2 and 3. In H. gon_1, Group A contained 285,831 SNPs and INDELs(approximately 89.31% of all variants) with an average sequencing depth of 92.55×; Group B contained 34,198 SNPs and INDELs (10.69% of the total) with an average depth of 33.67×. Within Group B, Group C comprised 4,989 loci (1.56% of the total) with an average depth of 43.36×, while Group D included 29,209 loci (9.13% of the total) with an average depth of 32.02×. T-test analyses indicated that the sequencing depth of Group A was significantly higher than that of Group B (*p* < 2.2e-16), and that Group C had a significantly higher depth than Group D (*p* = 2.186e-13). These statistical results confirm our hypothesis regarding the origins of the homozygous calls in the hybrid transcriptome data.

#### Identification of TN_D1 individuals

**Fig. 4:**
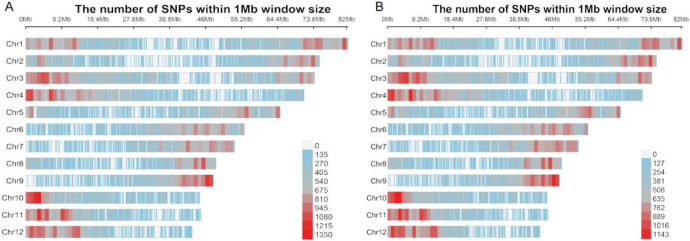
(**A**) Heterozygous SNP distribution of TN_D1 individuals. (**B**) Distribution of 257,140 randomly extracted SNPs from TN_D1 individuals.

**Fig. 5:**
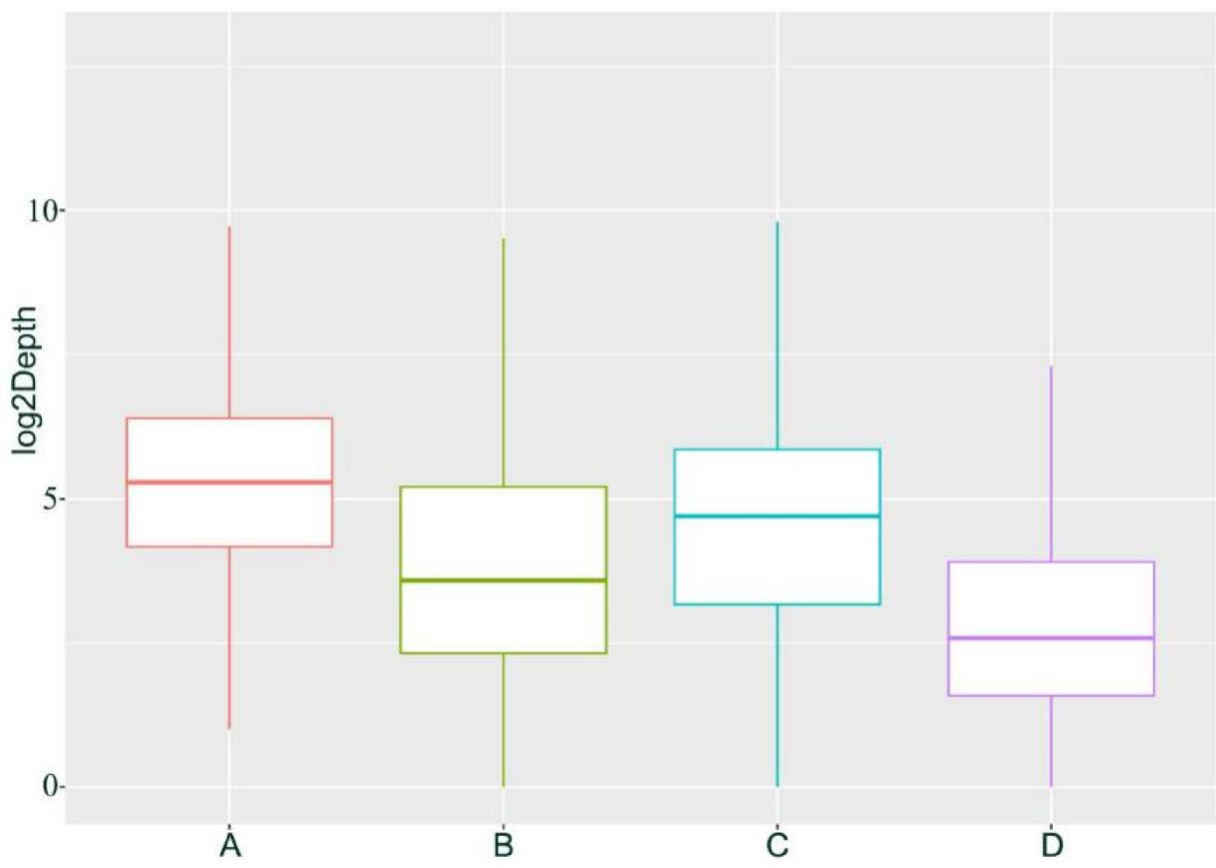
Depth distribution of SNPs and INDELs in four groups of TN_D1 individuals.

**Fig. 6:**
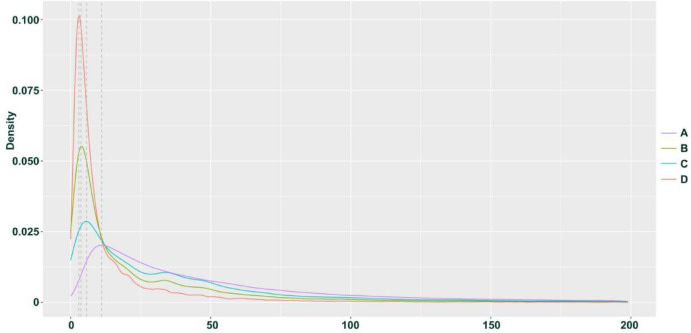
Density distribution of four groups of SNP depths in TN_D1 individuals.

We applied the same SNP calling and analysis approach to the hybrid offspring (TN_D) of *H*. *tibetana* and *H*. *neurocarpa* (Figs. 4). The results were similar to those observed in *H*. *goniocarpa*, confirming that all four heterozygous TN_D individuals are F1 hybrids. In samples collected at Da Ri (three individuals each of *H*. *tibetana* and *H*. *neurocarpa*), we identified 319,848 SNPs and INDELs that were consistently homozygous within each species but distinct between them. Among these, 257,140 loci (mean sequencing depth 97.37×) were heterozygous in TN_D1 individuals, representing approximately 80.39% of the total variants and corresponding to Group A as described for *H*. *goniocarpa*. The remaining 62,708 SNP loci, which are homozygous for a single parental allele, constitute Group B, with a mean sequencing depth of 43.95× (approximately 19.61% of the total). Within Group B, 32,757 SNP loci (mean depth 65.54×, ~ 10.24% of the total) are consistently homozygous and share the same genotype across all four individuals, defining Group C. The remaining 29,951 loci in Group B (mean depth 20.34×, ~ 9.36% of the total) form Group D. T-tests revealed that the sequencing depth for Group A was significantly higher than that for Group B (*p* < 2.2e-16), and similarly, Group C had a significantly higher depth than Group D (*p* < 2.2e-16). Box plots and density distributions illustrating the differences in SNP depth across these groups are provided in Figs. 5 and 6.

### Statistics of 8 individuals

Tables 4 and 5 summarize the SNP statistics for all eight individuals analyzed. Notably, the average sequencing depth for SNPs and INDELs in the TN_D batch is significantly higher than that in the *H*. *goniocarpa* batch. In contrast, the proportion of random error SNPs and INDELs (Group D) is significantly lower in the TN_D individuals compared to *H*. *goniocarpa*. The most striking difference between the batches is observed in ASE: TN_D1 exhibits more than 10% ASE SNPs (32,757 loci derived from 7,592 genes), whereas only about 1.5% ASE SNPs (4,989 loci derived from 1,683 genes) are detected in H. gon_1. This discrepancy likely reflects variations in gene expression due to differences in sampling periods between the two batches.

**Table 4:** Number of SNPs and indels per individual per group.

	A	B	C	D
H.gon_1	285,831(89.31%)	34,198(10.69%)	4989(1.56%)	29,209(9.13%)
H.gon_2	283,042(88.44%)	36,987(11.56%)	4989(1.56%)	31,998(10.00%)
H.gon_3	283,090(88.46%)	36,939(11.54%)	4989(1.56%)	31,950(9.98%)
H.gon_4	282,931(88.41%)	37,098(11.59%)	4989(1.56%)	32,109(10.03%)
TN_D1	257,140(80.39%)	62,708(19.61%)	32,757(10.24%)	29,951(9.36%)
TN_D2	259,557(81.15%)	60,291(18.85%)	32,757(10.24%)	27,534(8.61%)
TN_D3	263,290(82.32%)	56,558(17.68%)	32,757(10.24%)	23,801(7.44%)
TN_D4	262,358(82.03%)	57,490(17.97%)	32,757(10.24%)	24,733(7.73%)

**Table 5:** Mean depth of SNPs and indels per group.

	A	B	C	D	Average depth
H.gon_1	92.55	33.67	43.36	32.02	38.40
H.gon_2	79.23	27.66	37.57	26.11	33.51
H.gon_3	82.13	27.66	38.96	25.90	34.51
H.gon_4	85.37	30.74	40.66	29.20	35.98
TN_D1	97.37	43.95	65.54	20.34	39.61
TN_D2	95.01	44.32	63.95	20.96	38.81
TN_D3	96.35	49.04	67.24	24.00	40.11
TN_D4	94.66	47.10	65.90	22.21	39.62

### Gene expression profiles of 75 sea Buckthorn leaf samples

**Fig. 7:**
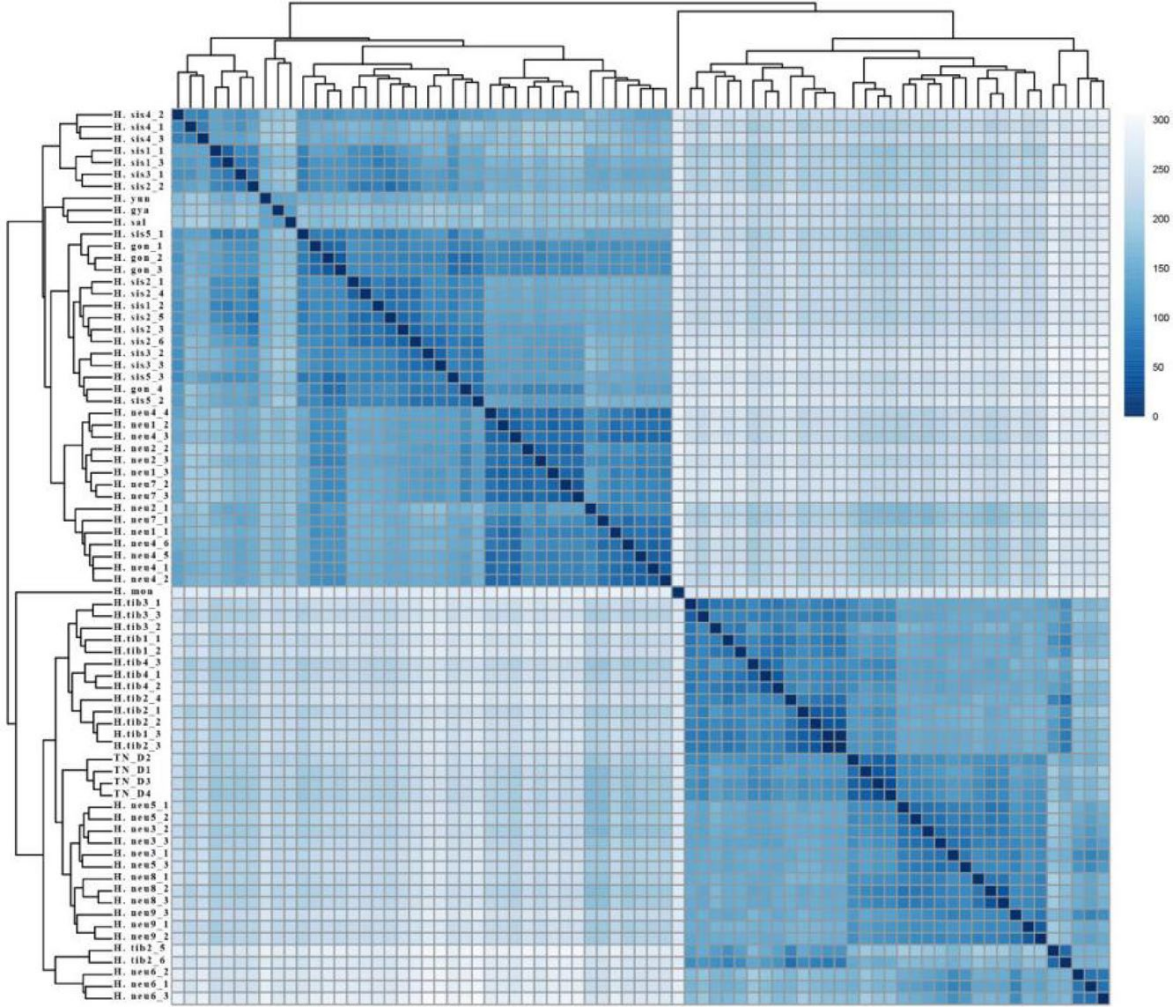
Correlation analysis of gene expression among 75 sea buckthorn leaf samples.

**Fig. 8:**
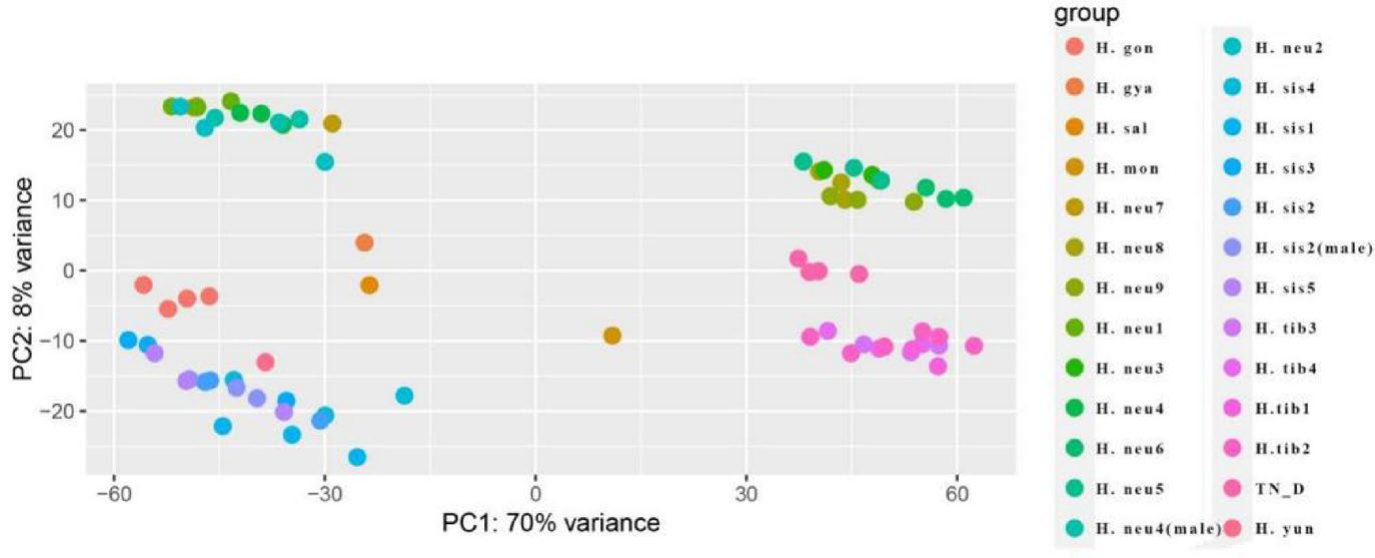
PCA analysis of 75 sea buckthorn leaf samples.

In the SNP calling results, *H*. *goniocarpa* and TN_D, both hybrid F1 generations, exhibited significant differences in the number of ASE genes. Various factors, such as the month of collection, time of day, and weather conditions on the sampling day, are speculated to contribute to these disparities in gene expression, as reflected in the transcriptome SNP calling results. A correlation analysis of gene expression across all sea buckthorn transcriptome data (Figs. 7 and 8) revealed that the clustering of gene expression data from these wild sea buckthorn samples did not strictly correspond to species classification. This finding suggests that differences in sample batches may contribute to gene expression variation, potentially affecting SNP calling outcomes.

### Phylogenomic analysis of sea Buckthorn

**Fig. 9:**
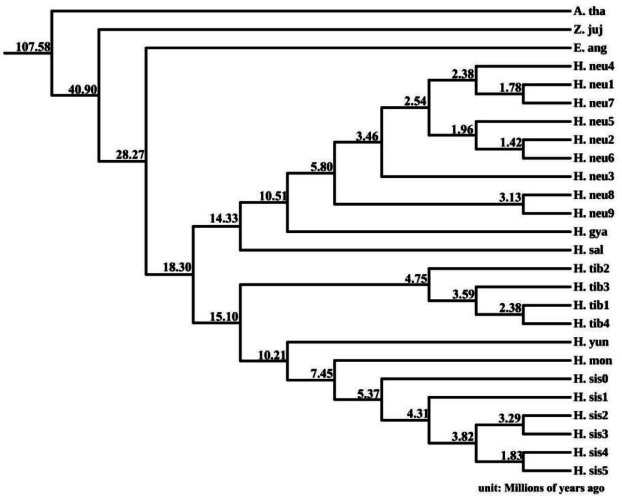
Phylogenomic tree. The figure includes *Arabidopsis thaliana* (A. tha), *Ziziphus jujuba* (Z. juj), *Elaeagnus angustifolia* (E. ang), and various sea buckthorn species (H. sis0 represents the reference genome, H. mon represents *H*. *rhamnoides subsp*. *mongolica*, H. yun represents *H*. *rhamnoides subsp*. *yunnanensis*, H. sal represents *H*. *salicifolia*, H. gya represents *H*. *gyantsensis*).

We constructed a phylogenomic tree using 101 single-copy orthologous genes from a set of eight species: *Arabidopsis*, *Ziziphus jujuba* Mill, *Elaeagnus moorcroftii* Wall, and seven sea buckthorn taxa (*H*. *rhamnoides ssp.*. *sinensis*, *H*. *rhamnoides subsp*. *mongolica*, *H*. *rhamnoides subsp*. *yunnanensis*, *H*. *tibetana*, *H*. *salicifolia*, *H*. *gyantsensis*, and *H*. *neurocarpa*), as illustrated in Fig. 9. The resulting tree clustered the sea buckthorn species into two distinct groups: one group comprised *H*. *rhamnoides ssp.*. *sinensis*, *H*. *rhamnoides subsp*. *mongolica*, *H*. *rhamnoides subsp*. *yunnanensis*, and *H*. *tibetana*, while the other group consisted of *H*. *neurocarpa*, *H*. *gyantsensis*, and *H*. *salicifolia*.

### Statistical analysis of genomic SNPs and indels

**Fig. 10:**
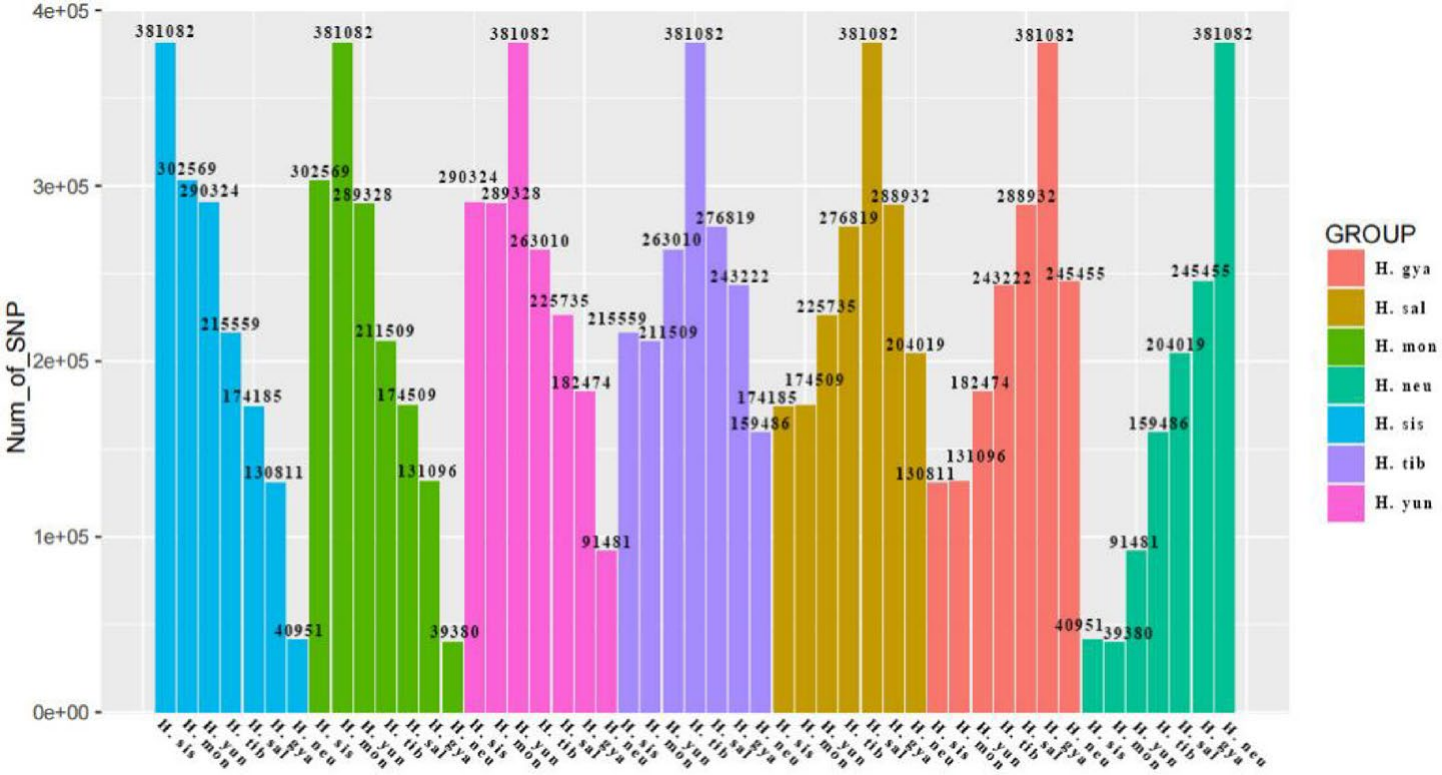
SNP consistency among seven sea buckthorn species. Each color represents a comparison between one focal species and the remaining six. SNPs are considered identical if both individuals share the same homozygous genotype or the same heterozygous genotype.

A total of 381,082 SNPs and INDELs were identified in the genomes of seven seabuckthorn individuals. In this study, H. sis5_1 was selected as the representative for *H*. *rhamnoides ssp.*. *sinensis*, H. tib4_1 for *H*. *tibetana*, and H. neu9_1 for *H*. *neurocarpa*. Pairwise comparisons among the individuals were conducted to enumerate loci with completely consistent SNPs and INDELs, as shown in Fig. 10.

**Fig. 11:**
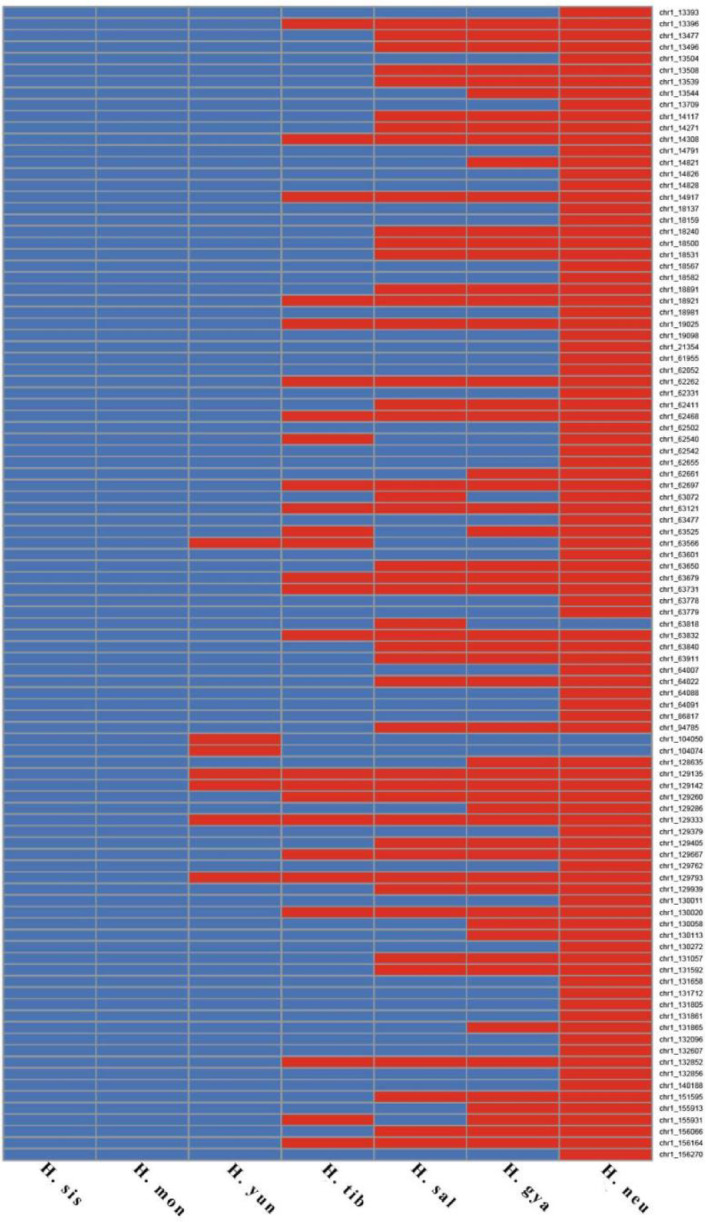
A subset of homozygous SNP and INDel loci on chromosome 1 among seven sea buckthorn individuals. All loci shown are homozygous and biallelic. For the same locus, identical colors indicate shared alleles among individuals.

**Fig. 12:**
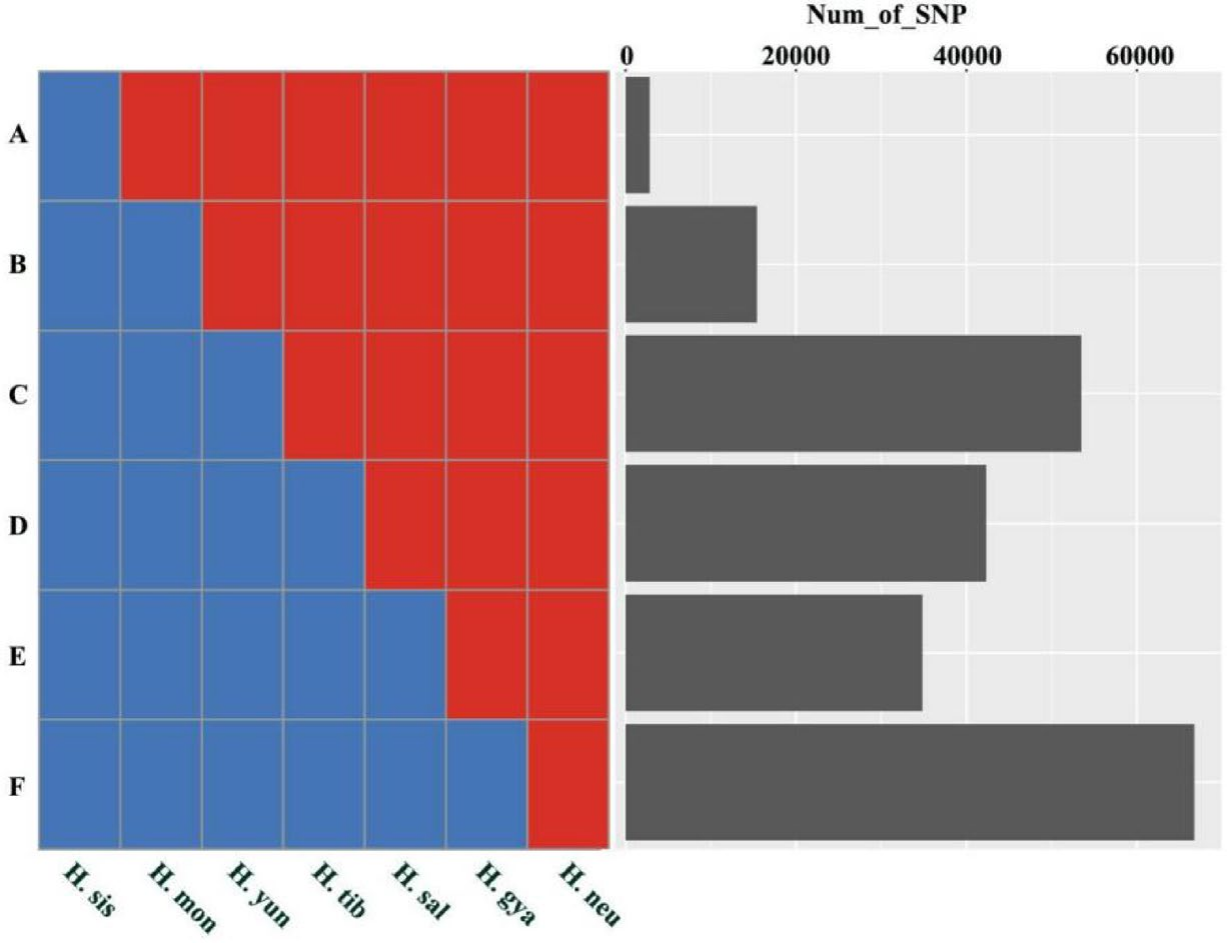
SNP and INDEL statistics. In this figure, the left panel corresponds to Fig. 11 and illustrates six types of SNP and INDEL distribution patterns. For example, type A represents variants specific to H.sis, distinguishing it from other types of sea buckthorn. Type B shows variants shared by H.sis and H.mon, which differentiate them from the remaining types. The right panel presents a bar chart summarizing the counts of each of the six SNP and INDEL types.

Our phylogenomic tree and molecular marker data (SNPs and INDELs) suggest that, following the divergence of two primary sea buckthorn lineages, subsequent population divergence led to the emergence of new species. This evolutionary process is supported by distinctive genomic features. If this scenario is correct, the genomes of the seven sea buckthorn species should form a continuum—with *H*. *rhamnoides ssp.*. *sinensis* and *H*. *neurocarpa* at the extremes and the other five species positioned between them. Accordingly, we conducted a statistical analysis of SNPs to assess genomic continuity. Of the 279,605 homozygous biallelic SNP loci examined, 215,651 (77.13%) exhibited continuity characteristics (Figs. 11 and 12; Table 6).

**Table 6:** Summary of six types of SNP and INDEL loci (see figure 12) identified in seven sea Buckthorn individuals.

GROUP	SNP_num	Percent	Cumulative percentage
A	2,823	1.01%	1.01%
B	15,437	5.52%	6.53%
C	53,464	19.12%	25.65%
D	42,325	15.14%	40.79%
E	34,850	12.46%	53.25%
F	66,752	23.87%	77.13%

## Discussion

Mapping tools such as HISAT2 and STAR^35^ are specifically designed for accurate transcriptome alignment and provide robust support for molecular marker analysis once transcriptome data are converted into BAM or SAM files. Although sequencing costs are relatively low, SNP calling from transcriptome data remains valuable, particularly when research materials are scarce, difficult to collect, or required for preliminary exploratory studies. In this study, we identified hybrid F1 generations using transcriptome data and highlighted the challenges associated with SNP calling.

Theoretically, an F1 hybrid should exhibit heterozygosity at most loci across all chromosomes. Detecting a high proportion of heterozygous sites supports the identification of a specimen as an F1 hybrid. In our transcriptome-based analysis of molecular markers, we observed heterozygosity rates ranging from 80.39 to 89.31%, which are notably high. Such levels of heterozygosity are highly unlikely in stabilized hybrid species. Even in cases involving backcrossing or inbred F2 generations, the heterozygosity rate typically decreases to approximately 50%, with about half of the homologous chromosomes becoming homozygous for one parental genotype. Therefore, the high and uniform distribution of heterozygous loci across chromosomes strongly supports the classification of the studied individuals as F1 hybrids.

In addition, 10.69–19.61% of the loci did not exhibit the expected heterozygosity. We speculate that these discrepancies are primarily caused by ASE and the low expression of certain genes. Indeed, previous studies have shown that ASE is a significant factor influencing SNP calling from transcriptomic data in F1 hybrids. For example, Shen et al.^36^ conducted pooled transcriptome sequencing on ten F1 hybrid individuals and assessed whether SNP-containing genes exhibited ASE by analyzing genotypes at loci showing differences between paternal and maternal lines. To minimize false positives due to random errors associated with low expression, they filtered out SNPs supported by fewer than 20 reads, aiming to identify genes with reliable allele-specific expression.

While Shen et al. aimed to identify ASE by analyzing F1 hybrids, our study focused on interpreting homozygous sites that deviated from the expected heterozygosity. In the H.gon_1 individual of *H. goniocarpa*, 10.69% of loci that were expected to be heterozygous based on parental genotypes appeared as homozygous. Of these, 9.13% were likely due to random errors associated with lowly expressed genes. Although the average sequencing depth at these loci was approximately 38×—a depth generally sufficient for accurate genotype calling in resequencing data—it may not reflect true coverage in transcriptome data. In RNA-seq, sequencing depth is influenced by gene expression levels rather than uniform genomic coverage. Nevertheless, we speculate that the actual error rate in identifying heterozygous sites from transcriptome data is likely lower than the observed percentage, largely due to our ability to distinguish between ASE and random sequencing errors.

In this study, we had access to only six parental individuals. When the three paternal individuals were homozygous for the genotype AA at a given SNP locus and the corresponding locus in the maternal individuals was homozygous for TT, we assumed that the genotype of the F1 hybrid should be heterozygous (AT). Using *H. goniocarpa* as an example, our results indicate that approximately 88.41–89.31% of the SNPs and INDELs in the four F1 hybrid individuals followed this expected inheritance pattern. Although three biological replicates are generally considered sufficient, there remains some uncertainty. The observation that all three parental individuals are homozygous at a locus does not guarantee that the true parents are also homozygous at that site. For loci appearing homozygous in the F1 hybrids due to low sequencing depth or ASE, we proposed the following criterion: if all four F1 individuals share the same homozygous genotype at a locus, and this genotype matches that of one parent, the locus may exhibit ASE. For example, if all paternal individuals are AA, all maternal individuals are TT, and all four F1 hybrids are TT, we interpret this as a case of ASE where only the maternal allele is expressed. In such cases, the observed genotype is likely due to the monoallelic expression of the maternal allele (Group C). Other mismatches are likely caused by low expression and considered random sequencing errors (Group D). We acknowledge that this approach is not rigorous, due to the limited number of individuals analyzed. Nonetheless, the clear difference in sequencing depth distributions between Groups C and D supports the reliability of our classification.

Historically, *H*. *goniocarpa* was considered an independent species; however, our analyses demonstrate that the four individuals originally identified as *H*. *goniocarpa* and the four TN_D hybrids are, in fact, F1 hybrids. Consequently, *H. goniocarpa* data were excluded from the construction of the phylogenomic tree. The resulting phylogeny is generally consistent with existing classifications of sea buckthorn, except for *H*. *tibetana* and *H*. *salicifolia*. Our results suggest that these two taxa may represent ancestral lineages, with an ancestral sea buckthorn diverging approximately 18.31 million years ago into two groups—one leading to *H*. *tibetana* and the other to *H*. *salicifolia*. Incomplete lineage sorting may explain discrepancies between morphological traits and the constructed phylogenomic tree. Subsequent adaptive divergence appears to have given rise to *H*. *rhamnoides subsp*. *yunnanensis*, followed by *H*. *rhamnoides subsp*. *mongolica* and *H. rhamnoides ssp. sinensis*, while another branch evolved into *H*. *salicifolia*, *H*. *gyantsensis*, and *H*. *neurocarpa*. It should be noted that while our dataset includes all known sea buckthorn species, it does not encompass all subspecies.

## Data Availability

The raw data of transcriptome sequencing for this project were deposited in the CNGB Nucleotide Sequence Archive (https://db.cngb.org/cnsa) and are accessible with the accession ID CNP0005649. H. rhamnoides subsp. Mongolica, H. rhamnoides subsp. yunnanensis, H. salicifolia, and H. gyantsensis can be respectively obtained from the SRA database using the sequence read archive accession numbers: ERR1294015, SRR17549372, SRR17549371, and SRR17549369.The assembled longest isoforms and the list of single-copy orthologous genes generated in this study are publicly available on Zenodo at: https://doi.org/10.5281/zenodo.15631788.
